# Poly(acrylic acid)-Sodium Alginate Superabsorbent Hydrogels Synthesized by Electron-Beam Irradiation—Part II: Swelling Kinetics and Absorption Behavior in Various Swelling Media

**DOI:** 10.3390/gels10090609

**Published:** 2024-09-23

**Authors:** Elena Manaila, Gabriela Craciun

**Affiliations:** Electron Accelerators Laboratory, National Institute for Laser, Plasma and Radiation Physics, 409 Atomistilor St., 077125 Magurele, Romania; elena.manaila@inflpr.ro

**Keywords:** poly(acrylic acid), sodium alginate, electron beam, superabsorbent hydrogels, swelling kinetics, absorption behavior

## Abstract

Hybrid hydrogels with superabsorbent properties based on acrylic acid (20%), sodium alginate (0.5%) and poly(ethylene oxide) (0.1%) were obtained by electron-beam irradiation between 5 and 20 kGy, and are characterized by different physical and chemical methods; the first results reported showed gel fractions over 87%, cross-link densities under 9.9 × 10^3^ mol/cm^3^ and swelling degrees of 400 g/g. Two types of hydrogels (without and with 0.1% initiator potassium persulfate) have been subjected to swelling and deswelling experiments in different swelling media with different pHs, chosen in accordance with the purpose for which these superabsorbent materials were obtained, i.e., water and nutrients carriers for agricultural purposes: 6.05 (distilled water), 7.66 (tap water), 5.40 (synthetic nutrient solution) and 7.45 (organic nutrient solution). Swelling kinetics and swelling dynamics have been also studied in order to investigate the influence of swelling media type and pH on the absorption phenomenon. The swelling and deswelling behaviors were influenced by the hydrogel characteristics and pH of the swelling media. Both the polymeric chain relaxation (non-Fickian diffusion) and macromolecular relaxation (super case II) phenomenon were highlighted as a function of swelling media type.

## 1. Introduction

Currently, new or with-improved-properties materials are needed more and more in fields like agriculture and environment [1,2], medicine [3,4,5,6] and engineering [5,7], their immediate availability being a necessity to adapt to the rapid technological progress and its consequences.

Among the promising polymer materials, a special class of materials has attracted wide interest in the last thirty years: hydrogels [8]. Hydrogels are cross-linked polymeric networks constituted by linear or branched hydrophilic polymers capable of retaining large amounts of water or biological fluids and maintaining at the same time the 3D hierarchical structures even in the swollen state [8,9,10]. To achieve the cross-linking, chemical or physical processes are required (e.g., radical polymerization, reaction of complementary groups, grafting reactions, etc.) [8,11]. Based on the composition and derivation, hydrogels can be categorized into synthetic (organic and inorganic), natural and hybrid [9,12]. Superabsorbent hydrogels are polymeric materials three-dimensionally cross-linked, linear or branched, with excellent hydrophilic properties, that are able to absorb large quantities of water or other liquids compared with general absorbing materials [13,14,15]. Regardless of the raw material nature (synthetic monomers or natural polymers), the superabsorbent obtaining method is the one that leads to their properties: physical bonds (non-covalent, temporary bonds), chemical (covalent, permanent bonds) or combined in the matrix structure [16]. The presence of physical bonds in the polymer matrix is specific to polymer materials with reversible properties, but with a short life and poor mechanical properties. Through chemical bonds, permanent hydrogels are obtained, with a high mechanical and thermal stability and controllable properties, ideal for applications that involve fast swelling and the slow release of water or other liquids, such as agriculture [17]. Besides chemical and physical methods, which use cross-linkers and complexing agents, respectively, irradiation by means of gamma radiation or electron beams represents a viable and attractive method for the obtaining of polymeric materials [18,19,20,21,22]. Electron-beam irradiation is a cheap, readily available, fast, reproducible and easily scaled-up method for the cross-linking, grafting or degradation of polymeric materials [23]. Electron-beam irradiation generally leads to cross-linking and to the obtaining of insoluble cross-linked materials in an aqueous media, being significantly more efficient than γ irradiation at the same irradiation dose delivered [23]. The nature of the material subjected to irradiation and the irradiation dose determine the occurrence of cross-linking and/or the grafting phenomena followed by degradation, if the irradiation continues. Thus, the irradiation gives the specific structure of the material and also changes it according with the purpose.

Sodium alginate or the sodium salt of alginic acid is a natural polymer extracted mainly from brown algae. It is a linear polysaccharide and exists in the form of a copolymer consisting of L-guluronic and D-mannuronic acid groups linked by 1,4-glycosidic bonds; it is often used to obtain durable, non-toxic hydrogels with a high degree of biodegradability, being ideal for applications in the agricultural, pharmaceutical or food sectors [22,24]. Hydrogels based on biopolymers such as sodium alginate have limited hardness and poor mechanical properties, these being major obstacles for their application on a large scale [25]. On the other hand, although synthetic superabsorbent polymeric materials are mechanically more robust compared to those based on biopolymers, they are less hydrophilic than them [17]. Therefore, in the recent years, studies involve the simultaneous radical polymerization of the monomer, cross-linking of the polymer and grafting of the resulting polymer onto sodium alginate molecules [26]. With this purpose, many studies published so far have reported the use of poly(acrylic acid-acrylamide) copolymers [27], poly(acrylic acid) [28] or poly(vinyl alcohol) (PVA) [29]. Polyacrylic acid is non-toxic and non-irritating, as compared with other monomers widely used for the synthesis of hydrogels. The advantages of the polyacrylic acid matrix over other hydrogels are related to its exceptional sensitivity to changes in the pH of swelling media [30,31]. Poly(ethylene oxide) (PEO) is a very good delivery vehicle, non-toxic and soluble in water, and can be used in the synthesis of hybrid hydrogels with superabsorbent properties. The ether oxygen from PEO forms hydrogen bonds with the hydroxyl groups from the sodium alginate that facilitate a better blending between them. Also, the addition of PEO to obtain cross-linked hydrogels by radiation techniques improves their physical and mechanical properties [32,33,34].

As a second part of this study in which hybrid hydrogels with superabsorbent properties were obtained by electron-beam irradiation and characterized by different physical and chemical methods [35], we propose to present here the results regarding the swelling kinetics and swelling dynamics in various swelling media (distilled and tap water and two nutrient solutions) of materials obtained and characterized in the previous study [35]. The hydrogel structure is of a cross-linked material, and the differences highlighted by FTIR analysis were attributed to the presence of the initiator–potassium persulfate (PP) reaction in different concentrations [35]. By SEM investigations, a homogeneous inner structure with pores and macropores with a slice-like aspect has been found. Differences in pore size and aspect were registered due to the irradiation dose and PP amount [35].

Therefore, hydrogels based on acrylic acid, sodium alginate and poly(ethylene oxide) obtained in the dose range of 5 to 20 kGy with and without the initiator–potassium persulfate (PP) reaction were used in swelling and deswelling experiments in four swelling media with different pHs (distilled water, tap water and two nutrient solutions used in agricultural applications) in order to investigate the influence of swelling media type and pH on the absorption phenomenon.

## 2. Results and Discussion

### 2.1. Swelling Experiment Results

Swelling studies have been performed on the hybrid hydrogels obtained by electron-beam irradiation in the range of 5 to 20 kGy without PP (called here Type I) and with 0.1% PP (called here Type II) [35], by immersion in four swelling media: distilled water (pH = 6.05), tap water (pH = 7.66), a synthetic nutrient solution—sol. A (pH = 5.40) and an organic one—natural bio-humus—sol. B (pH = 7.45). The swelling media were chosen based on the final purpose of the hydrogels obtained: water and nutrient delivery systems in real environments (for agricultural applications).

**Figure 1:**
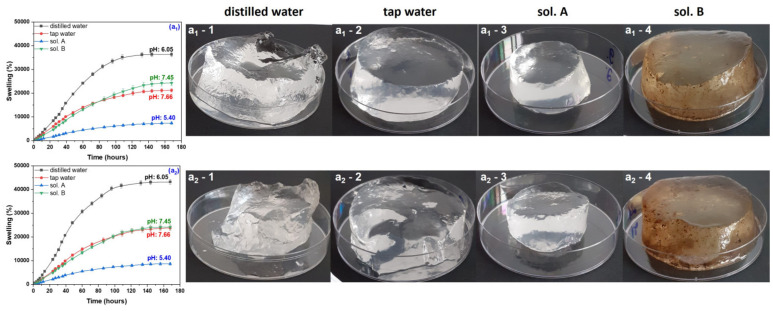
Swelling degree as a function of immersion time and images of Type I (**a_1_**) and Type II (**a_2_**) hydrogels obtained at 5 kGy and swollen in distilled water (**a_1_-1**,**a_2_-1**), tap water (**a_1_-2**,**a_2_-2**), sol. A (**a_1_-3**,**a_2_-3**) and sol. B (**a_1_-4**,**a_2_-4**).

**Figure 2:**
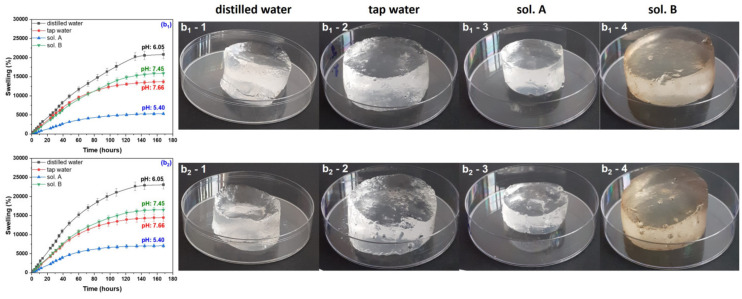
Swelling degree as a function of immersion time and images of Type I (**b_1_**) and Type II (**b_2_**) hydrogels obtained at 10 kGy and swollen in distilled water (**b_1_-1**,**b_2_-1**), tap water (**b_1_-2**,**b_2_-2**), sol. A (**b_1_-3**,**b_2_-3**) and sol. B (**b_1_-4**,**b_2_-4**).

**Figure 3:**
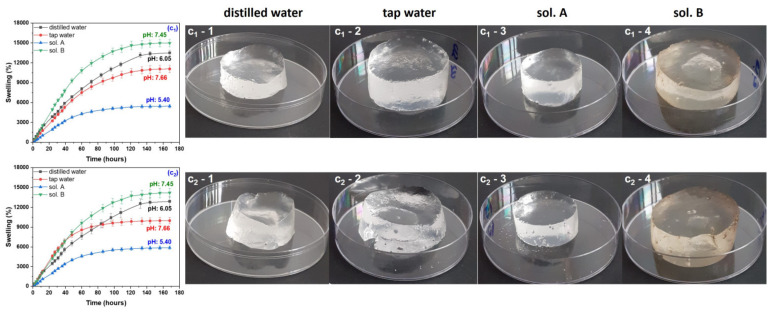
Swelling degree as a function of immersion time and images of Type I (**c_1_**) and Type II (**c_2_**) hydrogels obtained at 15 kGy and swollen in distilled water (**c_1_-1**,**c_2_-1**), tap water (**c_1_-2**,**c_2_-2**), sol. A (**c_1_-3**,**c_2_-3**) and sol. B (**c_1_-4**,**c_2_-4**).

**Figure 4:**
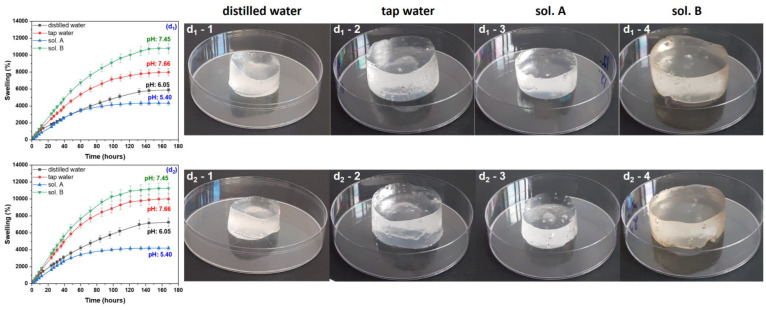
Swelling degree as a function of immersion time and images of Type I (**d_1_**) and Type II (**d_2_**) hydrogels obtained at 20 kGy and swollen in distilled water (**d_1_-1**,**d_2_-1**), tap water (**d_1_-2**,**d_2_-2**), sol. A (**d_1_-3**,**d_2_-3**) and sol. B (**d_1_-4**,**d_2_-4**).

During the swelling experiments, hydrogels have been taken out from the swelling media at various time intervals and weighed after removing the excess water by lightly dabbing the surfaces with paper napkins. All hydrogels used in the swelling experiments have been kept for 180 h in the swelling media, this being the time limit from which no absorption was registered. The swelling degree has been calculated and the results, together with images of hydrogels in the swollen state, are presented in Figure 1, Figure 2, Figure 3 and Figure 4. Data presented are the mean values of triplicate measurements. In order to appreciate the dimensions of the hydrogels shown in the images in Figure 1, Figure 2, Figure 3 and Figure 4, we specify that the polymeric materials were placed in transparent polystyrene Petri dishes with a diameter of 90 mm and a height of 14.2 mm.

As seen from the images in Figure 1, Figure 2, Figure 3 and Figure 4, as the irradiation dose has increased the hydrogels have become more and more stable in the swollen state. This can be correlated with previous results [35] where, in the same dose range, the gel fraction has increased by 0.09-times and the cross-link density by 23-times for hydrogels of Type I and by 18-times for hydrogels of Type II, respectively. These results are correlated with the cross-linking process in the radiation field and are attributed to the appearance of a large number of free radical active sites on the sodium alginate chains due to the irradiation dose increasing, favorable to the formation of a large number of acrylic acid graft bonds on the biopolymer chain [35].

The swelling media pH was also important, and its influence is to be discussed further. The swelling degree of all hydrogels (with and without PP) immersed in the acidic media (sol. A) were the lowest compared to that determined in all other swelling media.

Hydrogels obtained at the irradiation doses of 5 and 10 kGy, immersed in distilled water (on the border between acid and neutral), have showed the highest degrees of swelling: 40,000% and 20,000%, respectively. As the irradiation dose has increased, the swelling degrees were lower and lower in distilled water, above 12,000% for the samples without PP and below 12,000% for those with PP. For both types of hydrogels, at the irradiation dose of 20 kGy, the swelling degrees have been under 6000%.

Immersion in the neutral swelling media (sol. B and tap water) has led to degrees of swelling of 20,000% and over 12,000% for the hydrogels obtained at 5 and 10 kGy, respectively. The highest degrees of swelling in neutral solutions have been over 10,000% in sol. B and 9000% in tap water, respectively, for the hydrogels obtained at 20 kGy. No notable differences were recorded due to the presence of the reaction initiator in the hydrogels immersed in the neutral solutions.

**Figure 5:**
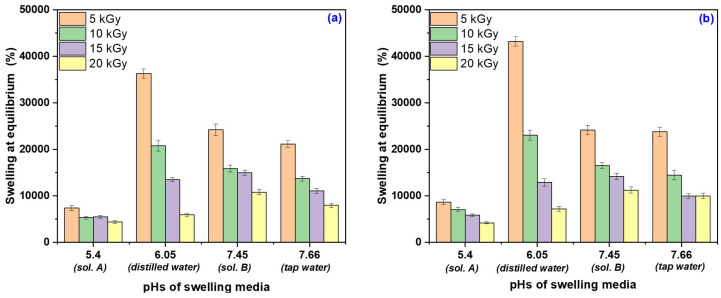
Swelling at equilibrium as a function of swelling media pHs for hydrogels of Type I (**a**) and Type II (**b**).

The results regarding the reaching of the equilibrium state as a result of the maximum liquid absorption (swelling at equilibrium) and the influence of swelling media pHs are presented in Figure 5.

As seen in Figure 5, swelling depends on both the pH of the swelling media and the hydrogel type. Those obtained at the irradiation dose of 5 kGy have showed the biggest absorption but were at the same time the most reactive to the pH changing. Thus, it can be said that the presence of PP influences the degree of swelling, but hydrogels are most sensitive to the pH of the swelling media, which is a measure of acidity or basicity of the solution through the hydrogen ions (H^+^). Swelling at equilibrium increases with the swelling media pH from 5.4 to 6.06 and the further increase of the pH from 7.45 to 7.66 leads to its decrease.

Tap water, sol. A and sol. B contain a variety of minerals and chemical substances dissociated as ionic compounds. Anions and cations that are formed induce in the swelling media a certain ionic strength (a measure of the total concentration of ions in the solution, dependent on the concentration and the charge of the ions), which also influences the degree of swelling.

Distilled water at the lower limit of the neutral zone absorbs atmospheric gases (including CO_2_) [36,37]. On the other hand, there are differences between the affinities of the micro- (fluorine, iodine, zinc, etc.) and macro-elements in them (calcium salts, magnesium, potassium, chlorides, nitrites, nitrates, etc.) from the tap water for the constituents of the hydrogel, especially for sodium alginate [36]. The swelling of hydrogels is due to the presence of strongly hydrophilic groups in the structure, namely carboxyl (–COOH) and hydroxyl (–OH) groups [37]. The protonation degree of the carboxylic groups (–COOH) of hydrogels, closely related to the swelling medium pH, gives the hydrogel its pH response (if the carboxylic groups are negatively charged they become more extended, facilitating the diffusion of water molecules in the hydrogel network, or if they are less polar they have, as a result, a low affinity with water) [36,38,39].

Since most of the carboxylic groups are negatively charged, they become more and more extended as the pH of the swelling media increases, which facilitates the diffusion of water molecules into the hydrogel network. Hydroxyl groups are mostly in protonated form and have a less polar character; therefore, at a low pH the polymer has a lower affinity with water and therefore a lower degree of swelling [39]. The presence of cations such as Na^+^, K^+^, Mg^2+^ and Ca^2+^, that are found in both tap water and nutrient solutions, influences the absorption behavior of the hydrogels.

The degree of swelling in sol. A is the lowest. This results from a charge shielding effect of the additional cations that causes an anion–anion electrostatic repulsion, and that leads to a decrease in the osmotic pressure difference between the polymer network and the external solution. At a given ionic strength, bivalent cations such as Mg^2+^ and Ca^2+^ contribute more charge than monocovalent cations Na^+^ and K^+^ and induce a greater decrease in intermolecular repulsion and an increased intermolecular interaction, which in turn leads to a great collapse of the hydrogel. In addition, divalent cations can chelate the carboxylic group, leading to a compact network and causing further contraction of the hydrogel [39]. The presence of Cl^−^, NO^2−^ and NO^3−^ anions in tap water makes the water molecule in the swelling medium serve as a hydrogen bond acceptor. The hydration of hydrogen bonds is stabilized by anionic hydration due to the improvement in the donation of water electron pairs [39], which is the reason why the degree of swelling in tap water is higher than in Sol. A.

Therefore, the type of ions present in the system influences the formation of hydrogen bonds with different strengths, a fact that can be explained based on the hydrogen bond hydration model of the polymer side chains [39,40]. According to this model, ion-specific swelling behavior is caused by the stabilization or destabilization of hydrogen bond hydration by ionic hydration. In the case of hydration to anions, the positive charge of water oxygen decreases and the negative charge of water oxygen increases. These changes in the hydrogen and oxygen charges in the swelling medium due to ion hydration correspond to a decrease in the electron pair accepting capacity (A-type) and an increase in the electron pair donating capacity (B-type), respectively. The presence of cations in the swelling media improves the hydrogen bond strength of the hydrogel because more electrical charges are pushed to the oxygen molecules between the O–H bond in the water molecule. Thus, the ability of the water hydrogen atom to accept the electrical pairs donated by the oxygen atom of the hydrogel increases and the hydrogen bonding of the hydrogel also increases. In this way, the A-type hydrogen bonding is stabilized. At the same time, the existence of anions makes the water molecule serve as a hydrogen bond acceptor. B-type hydrogen-bonded hydration is stabilized by hydration to the anion due to enhanced electron pair donation of water [39,40]. Similar results have been reported by other authors [41,42].

Based on the results obtained, it can be concluded that hydrogels can be used in agriculture (by vegetable growers in protected areas or in the field, plant growing units, seed and seedling producers) as support materials for fixing water and fertilizers, if the swelling media used are neutral and/or basic, but not acidic.

The swelling phenomenon of the superabsorbent is significantly influenced by the properties of the swelling media, including their charge valences and ionic strength [21]. In these conditions, for the cross-linked polymer, we determined the absorption at equilibrium (maximum absorbency) of the swelling media, Q (g/g), using also the elasticity gel theory of Flory’s [43,44,45]. The values of the charge density of polymer and the maximum absorbency Q (g/g) based on Flory’s equation are presented in Table 1 and Table 2.

The maximum water absorbency using Flory’s equation is controlled by the cross-link density, charge density and external ionic strength [45]. The difference between the values indicated in Table 1 can be associated with the waste of the cross-linker PP used for the hydrogel preparation, in ineffective cross-links and various topological defects such as cyclization, multiple cross-linking, loops or dangling ends [46,47]. Table 2 shows the change in hydrogel absorption performance induced by variation in the cross-linking degree and ionic strength and ion concentrations in swelling media. For the ionic strength S the following values were obtained: for tap water, 23.68 mM (50.02% cations and 49.98% anions); for sol. A, 23.87 mM (50.25% cations and 49.75% anions); and for sol. B, 23.88 mM (50.43% cations and 49.57% anions). The increasing of the cross-linking degree led to a decrease in the absorption of any of the swelling media used. The lowest values were obtained for sol. A, which has the lowest pH. Higher values were obtained for swelling media with a pH > 7 (tap water and sol. B), results that correlate with those obtained in the swelling experiments. The different concentrations of ions in the tap water, sol. A and sol. B also influenced the absorption capacity of the hydrogels. Usually, as the cation’s charge enhances, the hydrogel’s absorption capacity decreases, as was obtained in the case of tap water and sol. A. Although sol. B has the highest ionic charge, the absorption capacity of the hydrogel in it has increased. The result can be associated with the organic nature of the sol. B, being known to increase the swelling capacity in environments of this type [47,48].

### 2.2. Deswelling Experiment Results

**Figure 6:**
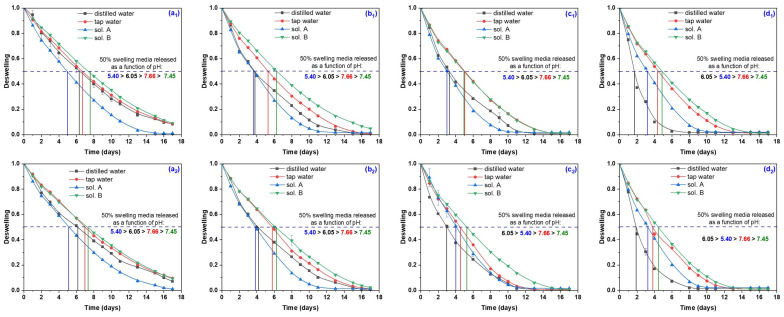
Deswelling degree of hydrogels obtained at 5 kGy—Type I (**a_1_**) and Type II (**a_2_**), 10 kGy—Type I (**b_1_**) and Type II (**b_2_**), 15 kGy—Type I (**c_1_**) and Type II (**c_2_**) and 20 kGy—Type I (**d_1_**) and Type II (**d_2_**).

The deswelling behaviors in time of all hydrogels in four swelling media (distilled and tap water and two nutrient solutions) are presented in Figure 6.

The influence of the irradiation dose at which the hydrogels were obtained can be observed. Thus, the amount of water and nutrient solutions absorbed at equilibrium is smaller and implicitly the loss of water is faster the higher the degree of cross-linking and the smaller the mesh size. It can be seen that hydrogels obtained at 10 kGy and 15 kGy have retained 46.5/66.2% and, respectively, 37.7/59.7% water or nutrient solutions after being kept at the ambient temperature of 23–25 °C for 4 days. Values are even higher for hydrogels of Type II. The slowest loss has been obtained for tap water (63.9% and 54.7% for hydrogels irradiated at 10 and 15 kGy) and sol. B (64.7% and 59.7% for the same hydrogels). After 6 days, the water retention ratio drops below 50%, reaching values of 36.1% and 44.4%, respectively, for tap water and sol. B (for hydrogels of Type II obtained at 15 kGy), the results being similar to those in the literature [49].

### 2.3. Swelling Kinetics

Swelling, the most important phenomenon in hydrogels, depends on various factors that affect the rate of swelling, such as the composition and structure of gel-forming polymers, cross-linking method, cross-linking degree, size of pores, number of hydrophilic functional groups, pH and concentration of swelling media or possible interaction between the particles in the swelling media and polymeric network [50,51,52,53,54,55,56,57,58,59,60,61,62,63]. Hydrogels swell in the swelling media and over time the swelling reaches a maximum value [50]. Various kinetics models, based on diffusion [50,64,65,66,67,68] or relaxation [50,64,65,66,67,68], have been suggested for appreciating the hydrogel swelling.

The main three steps involved in the swelling of hydrogels are the diffusion of water molecules into the polymer network, followed by the polymeric chain segment hydration and relaxation and finally the network expansion [50,61].

Because the first-order model is adequate for long periods of swelling, it was extensively used for modeling the swelling kinetics in the initial extent phase when the process is controlled by diffusion; however, it cannot be used for modeling the whole extent of swelling. When the swelling process is described by the relaxation and hydration of polymeric chains, the second-order kinetics model is widely applied for the modeling of swelling kinetics [50,67,68]. It should be noted that the second-order model depends directly on the hydrogel swelling capacity being still available over time and on the internal specific boundary area, these being closely related to the sites of the polymer network with a swelling capability that have not yet been hydrated by the solvent molecules [50,60,65,67,68,69].

**Figure 7:**
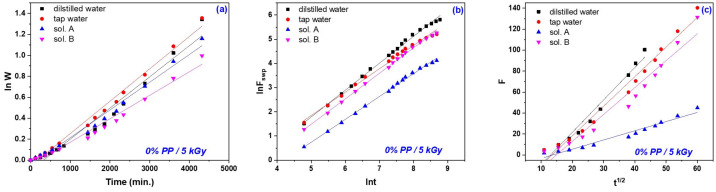
The first- (**a**) and second-order kinetics (**b**) and diffusional coefficient (**c**) determined for hydrogels of Type I, obtained at 5 kGy and swollen in distilled water, tap water, sol. A and sol. B.

**Figure 8:**
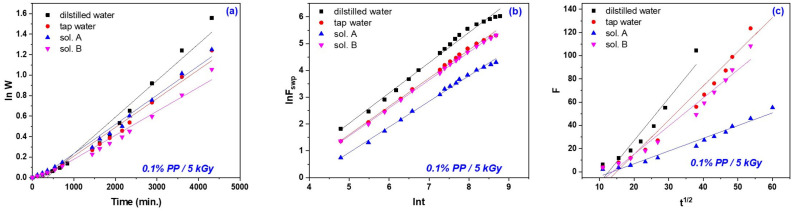
The first- (**a**) and second-order kinetics (**b**) and diffusional coefficient (**c**) determined for hydrogels of Type II, obtained at 5 kGy and swollen in distilled water, tap water, sol. A and sol. B.

The currently applied mathematical methods for describing the hydrogel behavior in aqueous liquids consider either diffusion or a combination of relaxation and diffusion but do not account for the polymer degeneration/degradation [70,71]. In these conditions, the first- and second-order kinetics based on the swelling equilibrium degree were applied in order to investigate the mechanisms involved in the swelling processes that took place in the four swelling media taken into account (distilled and tap water and nutrient solutions A and B) and the results are presented in Figure 7 and Figure 8 and Table 3, Table 4, Table 5 and Table 6.

The four swelling media in which the experiments were carried out for this study of swelling kinetics had different pHs: distilled water = 6.05, tap water = 7.66, sol. A = 5.4 and sol. B = 7.45. This study was carried out to show the dependence of the specific diffusion mechanism on their types and pHs. As can be seen from Figure 7a and Figure 8a and Table 3, the values of the first-order swelling rate constants increase with the irradiation dose increasing for both types of hydrogels, excepting the cases of those obtained at 20 kGy immersed in sol. B and tap water, respectively. Figure 7b and Figure 8b and Table 4 show that the values of second-order swelling rate constants increase with the irradiation dose increasing. The exception is the hydrogels of Type I at 20 kGy in sol. B and Type II at 20 kGy in both sol. A and B. It looks like the diffusion of nutrient solutions in hydrogels obtained at 20 kGy, especially for those of Type II, is affected by the presence of nutrients (NO_3_^−^, NH_4_^+^, P_2_O_5_ and K_2_O), micronutrients (copper, zinc, iron and manganese) and organic matter.

Analyzing Figure 7c and Figure 8c and Table 5 and Table 6, the simultaneous manifestation of water diffusion through the pores and the polymeric chain relaxation phenomenon associated with values of *n* between 0.5 and 1 (hydrogels of both types in sol. A and sol. B, excepting those irradiated at 15 kGy) attributed to the non-Fickian diffusion can be observed (from the values of swelling exponents/constants and diffusional coefficients) [8,50]. When the phenomenon of macromolecular relaxation is involved, there is a direct relationship with the flexibility of hydrogel polymer chains with the transport of liquid in the network [8]. In the case of the other hydrogels used in the diffusion study experiments in the four types of swelling media, which presented n values greater than 1, it can be said that the diffusion mechanism is exclusively governed by macromolecular relaxation, which is called Super Case II. The results presented above show that outside the Fickian diffusion model the liquid diffusion into the hydrogel network takes place over 70% from the initial absorption [8,72,73]. Also, the non-Fickian diffusion profile is attributed to the protonation of the carboxylic group at a low pH (5.40), which can favor the formation of hydrogen bonds with the hydroxyl group and which can pull the polymer chains close to form a tight network, thus reducing the degree of swelling. As the pH increases, the poly(acrylic acid) in the hydrogel forms carboxylate ions, which cause repulsion between the molecular chains [39].

**Figure 9:**
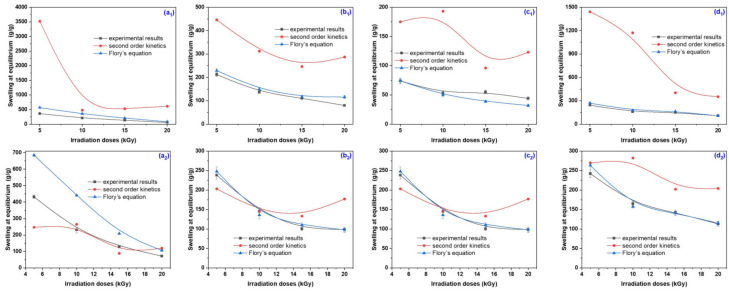
Comparative representation of swelling at equilibrium and maximum absorbency of swelling media of hydrogels obtained at 5 kGy—Type I (**a_1_**) and Type II (**a_2_**), 10 kGy—Type I (**b_1_**) and Type II (**b_2_**), 15 kGy—Type I (**c_1_**) and Type II (**c_2_**) and 20 kGy—Type I (**d_1_**) and Type II (**d_2_**).

The swelling at equilibrium and maximum water absorbency, determined based on the results of swelling over time (Figure 1, Figure 2, Figure 3 and Figure 4), the second-order equation (Table 4) and Flory’s equation (Table 1), are presented comparatively in Figure 9.

Figure 9 shows that although two different calculation methods were used to determine the same parameter (swelling at equilibrium/maximum absorbency), i.e., the equation for determining swelling at equilibrium *S_eq_* (Equation (2)), in which the masses of dried hydrogel and swollen hydrogels at time t and at equilibrium are used, and Flory’s equation (Equation (3)), in which the cross-link density, charge density and external ionic strength are used, the results obtained are comparable. We mention that all the physical quantities involved in the swelling at equilibrium, *S_eq_.*, and maximum absorbency, Q(g/g), are experimentally determined and represent the average of three measured values.

### 2.4. Scanning Electron Microscopy (SEM) Investigation

**Figure 10:**
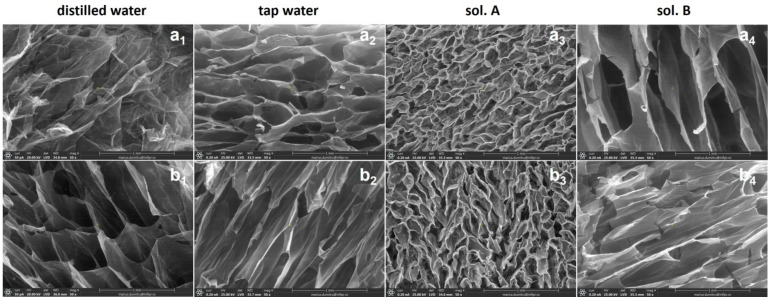
SEM micrographs of Type I (**a_1_**–**a_4_**) and Type II (**b_1_**–**b_4_**) hydrogels obtained at 5 kGy.

**Figure 11:**
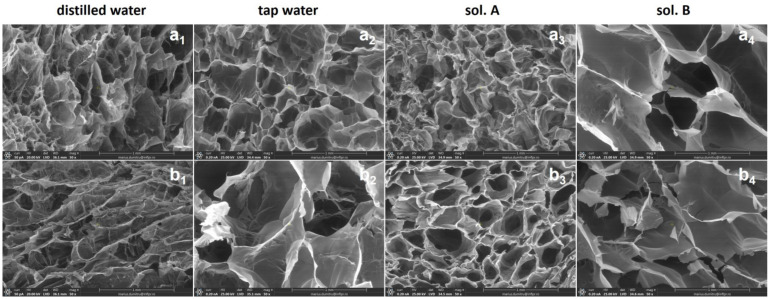
SEM micrographs of Type I (**a_1_**–**a_4_**) and Type II (**b_1_**–**b_4_**) hydrogels obtained at 20 kGy.

The inner morphology of hydrogels in freeze-drying state was investigated by Scanning Electron Microscopy (SEM). Images and details of hydrogels obtained at 5 and 20 kGy, with and without PP, after swelling in distilled water, tap water, sol. A and sol. B are presented in Figure 10 and Figure 11.

Differences between the inner structures of hydrogels without PP compared to that with PP can be observed in terms of pore sizes and shapes. The hydrogels of both types obtained at 5 kGy were quite unstable in the final swelling phase, their internal structure being, as shown in Figure 10, one with many pores but with rather thin walls.

As can be seen from Figure 11, the irradiation dose increasing has led to the obtaining of hydrogels with structures having a high and regular porosity and firm walls. Also, both the pH difference between the swelling media in which the swelling kinetics were studied and the structure of the polymer networks have influenced the diffusion process (at least in its initial phase). Thus, immersion in swelling media with a more acidic pH (distilled water and sol. A), although it opened the pores thus allowing their diffusion in the polymer network, after lyophilization allowed the visualization of their very different sizes. Immersion in liquids with a more alkaline pH (tap water and sol. B) revealed a more regular pore network with larger sizes [74].

## 3. Conclusions

Swelling experiments performed with hybrid hydrogels, obtained by an electron beam of 5.5 MeV irradiation, in four swelling media (distilled water, tap water and two nutrient solutions) with different pHs have showed that the increasing of the irradiation dose leads to the obtaining of hydrogels increasingly stable in the swollen state. Immersion of hydrogels in acidic environment negatively influences their swelling degree. High swelling degrees, between 40,000% and 20,000%, are associated with the obtaining conditions (low irradiation doses) and immersion in neutral swelling media, tap water and organic nutrient solution.

The swelling kinetics studies showed that some hydrogels immersed in the nutrient solutions presented simultaneous manifestation of water diffusion through the pores and the polymeric chain relaxation phenomenon (non-Fickian diffusion), while others obtained especially at high irradiation doses swelled exclusively through the macromolecular relaxation (super case II).

The diffusion of nutrient solutions in hydrogels obtained at 20 kGy, especially for those containing the reaction initiator, was affected by the presence of nutrients (NO_3_^-^, NH_4_^+^, P_2_O_5_ and K_2_O), micronutrients (copper, zinc, iron and manganese) and organic matter.

The use of two different calculation methods to determine the same parameter (swelling at equilibrium/maximum absorbency), i.e., the equation for determining swelling at equilibrium *S_eq_* and Flory’s equation, generates comparable results and confirm the experimental data.

The increasing of the cross-linking degree led to a decrease in the absorption of any swelling media used. The lowest absorptions were obtained after immersion in the synthetic nutrient solution with the lowest pH. Higher values were obtained for swelling media with a pH > 7 (tap water and organic nutrient solution), results that are in correlation with those obtained in the swelling experiments.

## 4. Materials and Methods

### 4.1. Materials

The materials used in the experiments are hydrogels obtained by electron-beam irradiation in the range of 5 to 20 kGy based on one recipe presented in a previous work [35,75]. ALID 7, the linear accelerator of 5.5 MeV built in the Electron Accelerators Laboratory from the National Institute for Lasers, Plasma and Radiation Physics, Bucharest, Romania, was used as the radiation source. Radiation dosimetry was performed using a graphite calorimeter, provided by DTU Health Tech, High Dose Reference Laboratory, Roskilde, Denmark, which is the primary standard for electron beams [35,75].

Hydrogels obtaining chemicals purchased from Merck KGaA, Darmstadt, Germany have been used without any further modification, with the following characteristics given by the producer: acrylic acid (M_w_ = 71.08 g/mol, density = 1.13 g/cm^3^) was used in a percentage of 20%, sodium alginate (M_w_ = 120,000–190,000 g/mol, viscosity = 15–25 cP, 1% in water) in a percentage of 0.5%, potassium persulfate K_2_S_2_O_8_ (M_w_ = 270.322 g/mol, density = 2.477 g/cm^3^) as a reaction initiator in a percentage of maximum 0.1% and poly(ethylene oxide (M_w_ = 300,000 g/mol, density = 1.210 g/cm^3^) in a percentage of 0.1% [35,75].

Two types of hybrid hydrogels, without (type I) and with (type II) potassium persulfate, were characterized as in the previous work [35,75] before being used and their specific properties are presented in Table 7.

Swelling experiments have been performed in two types of water (distilled and tap) and in two nutrient solutions (sol. A and sol. B). The distilled water has been obtained using a laboratory double distiller, the tap water was of potable type from the Magurele, Romania, town network and the nutrient solutions were bought from a special store in Romania. Specifications of distilled water, tap water and nutrient solutions used in the swelling experiments are presented in Table 8.

### 4.2. Methods

#### 4.2.1. Swelling and Deswelling Experiments

The hydrogel swelling dynamic was studied in distilled and tap water, sol. A and sol. B at the room temperature of 25 °C, using 0.2 g of dry hydrogel each. The increasing of hydrogel mass was evaluated by regular weighing that has been carried out as follows: in the first 48 h carried out at approximately equal time intervals (12 weighing), then twice a day (every 12 h) at the same hours. It was considered that the equilibrium has been reached when, from one day to the next one, there were no more mass increases. The maximum maintenance time of the hydrogels in the swelling medium did not exceed 180 h. Once the equilibrium has been reached, the samples were taken out, dried in air for 6 days and finally dried in a vacuum oven for 48 h at 50 °C to constant weight [35,75,76,77,78].

Equations (1) and (2) have been used for the swelling *S*(%) and swelling at equilibrium *S_eq._* (%) calculation [35,75,76,77,78].

(1) $$S\left(\%\right)=\frac{W_{t}-W_{i}}{W_{i}}\times 100,$$

(2) $$S_{eq.}\left(\%\right)=\frac{W_{eq.}-W_{i}}{W_{i}}\times 100,$$

where *W_t_*, *W_eq_* and *W_i_* are the masses of swollen hydrogels at time, *t*, at equilibrium and in their initial dry state [35,75].

The maximum absorbency, *Q* (g/g), has been calculated using Equation (3) from the elasticity gel theory of Flory’s [43,44,45]:

(3) $$Q^{5/3}\approx \frac{{\left(\frac{i}{2V_{u}S^{1/2}}\right)}^{2}+\frac{\left(\frac{1}{2}-X_{1}\right)}{V_{1}}}{\frac{V_{E}}{V_{0}}},$$

where *Q* is the maximum water absorbency (g/g), *i/V_u_* is the charge density of polymer (*V_u_* is the volume of the structural unit and *i* is the electronic/ionic charge present on the polymer backbone per polymer unit), *S* is the ionic strength of the external solution (mol⋅L^−1^), (1/2 − *X*_1_)/*V*_1_ is the polymer–solvent affinity (*X*_1_: interaction parameter of polymer with solvent; *V*_1_: molar volume of solvent in a real network) and *V_E_*/*V*_0_ is the cross-linking density of polymer (amount cross-linked/total polymer).

The charge density of polymer *i*/*V_u_* (*f_eff_*) was calculated using the following equation [44,46]:

(4) $$ln\left(1-\nu_{2}\right)+\nu_{2}+\chi \nu_{2}+N^{-1}\left(\nu_{2}^{1/3}\nu_{2}^{{2/3}^{2/3}}-\nu_{2}/2\right)-f_{eff}\nu_{2}=0$$

where *χ* is the interaction parameter between the polymer network and water, *N* is the average network chain length between two successive cross-links and *υ*_2_ (cm^3^) is the volume fraction of the polymer in the swollen gel.

The ionic strength *S* of all solutions has been calculated using the following equation:

(5) $$S=\frac{1}{2}\sum_{i=1}^{n}c_{i}z_{i}^{2}$$

where *c* is the concentration of the dissolved salt ion in mol⋅L^−1^ and *z* is the valence of the ion. For the dissolved salts, a complete dissociation was assumed [45,47].

For deswelling investigations, hydrogels at swelling equilibrium were placed in Petri dishes at the laboratory temperature of 23–25 °C. Then, they were weighed daily for 10 days. Based on the mass loss, the change in the cross-linking degree was calculated. The deswelling degree was determined also from the mass loss in time, using the following equation [79]:

(6) $$Deswelling=\frac{m_{t}}{m_{eq.}},$$

where *m_t_* and *m_eq._* are the hydrogel masses at time *t* and at equilibrium.

#### 4.2.2. Swelling Kinetics Methodology

In order to investigate the mechanism of swelling and to verify experimental data, several kinetic models have been used. The number and arrays of the chemical groups from acrylic acid and sodium alginate chains imply that there are many types of polymer–solvent interactions and it is quite probable for any kinetic of swelling to be global [77].

A simple kinetic analysis is the first-order equation, Equation (7), which becomes Equation (8) after integration and by applying the initial condition (*S* = 0 at *t* = 0 and *S* = *S* at *t* = *t*) [77]:

(7) $$\frac{dS}{dt}=k_{1,S}\left(S_{max.}-S\right),$$

(8) $$lnW=k_{1,S}t,\;were\;W=\frac{S_{max.}}{S_{max.}-S},$$

where *k*_1,*S*_ is the rate constant of first-order swelling, *S* and *S_max._* are the degrees of swelling at time *t* and at equilibrium, respectively.

A second-order equation (Equation (9)) based on the degree of equilibrium can also be applied [77]. After integration and by applying the initial condition (*S* = 0 at *t* = 0 and *S* = *S* at *t* = *t*), it becomes Equation (10):

(9) $$\frac{dS}{dt}=k_{2,S}{\left(S_{max.}-S\right)}^{2},$$

(10) $$\frac{t}{S}=A+Bt,were\;A=r_{0}=\frac{1}{k_{2,S}\times S_{max.}^{2}\;}\;and\;B=\frac{1}{S_{max.}},$$

where *A = r_0_ =* 1/(*k*_2,*S*_*S_max._^2^*) is the initial swelling rate of the hydrogel, *k*_2,*S*_ is the swelling rate constant and *B =* (1/*S_max._*) is the inverse of the maximum or equilibrium swelling [77].

For both, first- and second-order swelling kinetics, ln*W* versus *t* and *t/S* versus *t*, respectively, were plotted [77]. From the slopes and intersections of plotted lines, the rate constant of first- and second-order swelling (*k*_1,*S*_ and, *k*_2,*S*_), the initial rate of swelling (*r*_0_) and the theoretical swelling at equilibrium (*S_max_*_._) values were determined [77].

By applying Equation (11), which becomes Equation (12) to 60% of the swelling curves, the nature of the diffusion or the swelling mechanism of water (or other aqueous solutions) into hydrogels was evaluated [80,81].

(11) $$S_{swp}=\frac{W_{t}-W_{0}}{W_{0}}=kt^{n},$$

(12) $$lnS_{swp}=nlnt+lnk\;,$$

where *S_swp_*, *W_t_* and *W*_0_ are the fraction swelling ratio at time *t*, the weight of the swollen hydrogels at time *t* and the weight of dry hydrogels, respectively, *k* is the swelling constant and *n* is the swelling exponent that indicates the water transport mechanism. From the slopes and intercepts in ln*S_swp_* versus ln*t* graphic, the swelling exponent (*n*) and swelling constant (*k*) values have been calculated.

The values of *n* determine the type of the water transport mechanism in accordance with Fick’s theory [82]. The Fickian diffusion (case I), associated with a low water mobility comparative with the polymeric chain movement, corresponds to a value of diffusion parameter *n* of 0.5. In this case, the equilibrium state is rapidly attained. Anomalous diffusion has associated values of *n* between 0.5 and 1; here, the water mobility is comparable with the movement of the polymeric chains. The non-Fickian diffusion (case II where *n* = 1 and super Case II where *n* > 1) corresponds to a rapid water mobility comparative with the polymeric chain relaxation [83,84].

The swelling-time curves of hydrogels in water were used to calculate the diffusion coefficients (D) by using Equation (13). The method is valid only for the first 60% of the swelling [77].

(13) $$S=4{\left[\frac{D}{\pi \times r^{2}}\right]}^{1/2}t^{1/2},$$

where S, *D*, *r* and *t* represent the swelling ratio, the diffusion coefficient and the radius of the hydrogel in a cylindrical form and time, respectively.

The diffusion coefficient value *D* was calculated from the *S* versus *t*^1/2^ graphic in which the slope of the line gives the diffusion coefficient value *D*.

#### 4.2.3. Morphological Investigations by Scanning Electron Microscopy

Lyophilized hydrogels have been examined by the Scanning Electron Microscopy (SEM) technique using the FEI/Phillips scanning electron microscope (Hillsboro, OR, USA) [35,36].

## Figures and Tables

**Table 1:** Values of polymer charge density.

Dose (kGy)	Distilled Water (pH = 6.05)	Tap Water (pH = 7.66)	Sol. A (pH = 5.40)	Sol. B (pH = 7.45)
0% PP (type I)				
5	0.910 ± 0.001	0.892 ± 0.001	0.820 ± 0.008	0.898 ± 0.002
10	0.824 ± 0.002	0.805 ± 0.002	0.714 ± 0.008	0.813 ± 0.002
15	0.813 ± 0.0028	0.802 ± 0.004	0.698 ± 0.009	0.822 ± 0.002
20	0.849 ± 0.009	0.934 ± 0.005	0.772 ± 0.015	0.925 ± 0.002
0.1% PP (type II)				
5	0.996 ± 0.001	0.975 ± 0.002	0.894 ± 0.008	0.976 ± 0.003
10	1.134 ± 0.003	1.084 ± 0.007	0.975 ± 0.014	1.096 ± 0.003
15	0.998 ± 0.004	0.979 ± 0.005	0.874 ± 0.010	0.999 ± 0.004
20	0.838 ± 0.010	0.877 ± 0.006	0.738 ± 0.013	0.888 ± 0.005

**Table 2:** Maximum absorbency Q (g/g) based on Flory’s equation.

Dose (kGy)	Distilled Water (pH = 6.05)	Tap Water (pH = 7.66)	Sol. A (pH = 5.40)	Sol. B (pH = 7.45)
0% PP (type I)				
5	567 ± 6	229 ± 8	74 ± 4	270 ± 10
10	353 ± 11	149 ± 6	50 ± 3	178 ± 6
15	207 ± 6	117 ± 6	39 ± 2	165 ± 5
20	84 ± 1	116 ± 7	32 ± 2	107 ± 4
0.1% PP (type II)				
5	685 ± 7	248 ± 11	84 ± 4	263 ± 11
10	441 ± 1	136 ± 10	55 ± 5	158 ± 4
15	209 ± 1	110 ± 6	50 ± 2	141 ± 5
20	106 ± 5	98 ± 7	32 ± 2	115 ± 5

**Table 3:** The first-order kinetics. First-order swelling rate constants (*k*_1,*S*_ × 10^4^/min^−1^).

Dose (kGy)	Distilled Water (pH = 6.05)		Tap Water (pH = 7.66)		Sol. A (pH = 5.40)		Sol. B (pH = 7.45)	
	*k*_1,*S*_	*R* ^2^	*k*_1,*S*_	*R* ^2^	*k*_1,*S*_	*R* ^2^	*k*_1,*S*_	*R* ^2^
	Hydrogels of Type I (0% PP)							
5	1.394	0.995	2.100	0.995	1.809	0.999	1.360	0.996
10	1.947	0.999	2.358	0.997	2.286	0.993	1.782	0.999
15	2.641	0.997	2.393	0.999	2.644	0.998	2.477	0.994
20	3.475	0.979	2.658	0.996	2.878	0.998	2.298	0.999
	Hydrogels of Type II (0.1% PP)							
5	1.288	0.994	1.530	0.996	1.911	0.998	1.435	0.996
10	1.977	0.997	2.096	0.997	2.437	0.989	2.080	0.998
15	2.266	0.996	3.441	0.996	2.542	0.996	2.134	0.999
20	2.887	0.990	2.432	0.995	3.030	0.992	2.384	0.990

**Table 4:** The second-order kinetics. Second-order swelling rate constants (*k*_2,*S*_ × 10^9^/g gel (g water min)^−1^) and equilibrium swelling (*S_eq_*/g water (g gel)^−1^).

Dose (kGy)	Distilled Water (pH = 6.05)			Tap Water (pH = 7.66)			Sol. A (pH = 5.40)			Sol. B (pH = 7.45)		
	*k*_2,*S*_	*S_eq_*	*R* ^2^	*k*_2,*S*_	*S_eq_*	*R* ^2^	*k*_2,*S*_	*S_eq_*	*R* ^2^	*k*_2,S_	*S_eq_*	*R* ^2^
	Hydrogels of Type I (0% PP)											
5	0.046	352,010	0.022	2.419	44,622	0.888	4.809	17,478	0.907	0.167	144,095	0.309
10	2.929	47,914	0.014	4.177	31,206	0.509	4.935	19,337	0.899	0.269	117,094	0.430
15	1.461	52,834	0.975	6.099	24,607	0.946	15.204	9635	0.941	1.853	40,321	0.922
20	1.289	61,253	0.790	4.359	28,678	0.890	13.884	12,290	0.997	3.003	35,204	0.909
	Hydrogels of Type II (0.1% PP)											
5	5.637	24,686	0.977	7.072	20,229	0.962	22.593	8869	0.934	5.780	26,850	0.930
10	4.202	26,542	0.981	19.458	14,574	0.929	17.990	9989	0.914	4.355	28,181	0.917
15	23.880	8820	0.935	13.178	13,249	0.979	32.532	6813	0.955	6.464	20,203	0.981
20	13.863	11,970	0.945	8.786	17,653	0.960	40.959	6273	0.955	7.301	20,359	0.952

**Table 5:** The values of swelling exponents and constants (*k* × 10^2^).

Dose (kGy)	Distilled Water (pH = 6.05)			Tap Water (pH = 7.66)			Sol. A (pH = 5.40)			Sol. B (pH = 7.45)		
	*k*	*n*	*R* ^2^	*k*	*n*	*R* ^2^	*k*	*n*	*R* ^2^	*k*	*n*	*R* ^2^
	Hydrogels of Type I (0% PP)											
5	2.228	1.130	0.999	3.326	1.035	0.999	1.910	0.939	0.999	2.366	1.045	0.999
10	5.873	0.936	0.998	4.261	0.950	0.998	2.710	0.867	0.991	3.463	0.963	1.000
15	6.849	0.884	0.999	4.393	0.909	0.999	1.770	0.960	0.992	3.523	0.997	0.997
20	10.405	0.725	0.998	5.051	0.852	0.997	1.846	0.913	0.998	4.037	0.913	0.999
	Hydrogels of Type II (0.1% PP)											
5	2.650	1.124	0.992	2.214	1.075	0.998	1.953	0.968	0.997	2.569	1.042	0.998
10	3.110	1.051	0.997	3.389	0.979	0.997	2.309	0.947	0.987	3.304	0.996	0.999
15	3.773	0.955	0.990	3.891	1.086	0.998	1.806	0.996	0.996	3.336	0.980	0.999
20	8.054	0.773	0.998	4.209	0.905	0.996	1.948	0.926	0.991	3.401	0.955	0.997

**Table 6:** The values of diffusional coefficient (*D* × 10^3^/cm^2^s^−1^).

Dose (kGy)	Distilled Water (pH = 6.05)		Tap Water (pH = 7.66)		Sol. A (pH = 5.40)		Sol. B (pH = 7.45)	
	*D*	*R* ^2^	*D*	*R* ^2^	*D*	*R* ^2^	*D*	*R* ^2^
	Hydrogels of Type I (0% PP)							
5	1.980	0.975	4.430	0.977	0.241	0.974	1.852	0.965
10	1.019	0.981	1.953	0.979	0.173	0.974	1.575	0.978
15	0.728	0.997	1.585	0.983	0.135	0.965	0.948	0.955
20	0.197	1.000	0.593	0.987	0.100	0.972	0.896	0.984
	Hydrogels of Type II (0.1% PP)							
5	2.297	0.958	4.617	0.965	0.845	0.969	3.277	0.968
10	1.465	0.950	2.854	0.972	0.834	0.975	2.319	0.978
15	1.060	0.991	2.265	0.987	0.464	0.976	1.650	0.982
20	0.329	0.995	0.948	0.982	0.278	0.981	1.268	0.820

**Table 7:** Physico-chemical properties of hydrogels used in swelling experiments: G, gel fraction (%); q, cross-link density (mol/cm^3^); ξ, mesh size (nm); and P, porosity (%).

Dose (kGy)	Type I (0 % PP)				Type II (0.1% PP)			
	G (%)	q × 10^3^ (mol/cm^3^)	ξ (nm)	P (%)	G(%)	q × 10^3^ (mol/cm^3^)	ξ (nm)	P (%)
5	87.78 ± 3.30	0.434 ± 0.02	223.1 ± 5.87	99.71 ± 0.01	88.86 ± 2.13	0.382 ± 0.01	248.6 ± 5.02	99.77 ± 0.01
10	93.39 ± 2.37	0.840 ± 0.07	138.8 ± 7.96	99.51 ± 0.02	91.79 ± 3.30	1.117 ± 0.11	121.3 ± 8.25	99.57 ± 0.03
15	94.07 ± 2.23	1.909 ± 0.09	78.3 ± 5.54	99.24 ± 0.04	92.97 ± 3.97	2.958 ± 0.24	60.4 ± 3.24	99.23 ± 0.03
20	94.48 ± 1.94	9.958 ± 0.82	25.6 ± 1.44	98.31 ± 0.09	94.56 ± 2.16	6.770 ± 0.86	33.3 ± 2.86	98.61 ± 0.10

**Table 8:** Specifications of distilled water, tap water and nutrient solutions (sol. A and sol. B).

Swelling Media	Specifications
Distilled water	Boiling point: 100 °C; freezing point: 0 °C; oxidizing capacity: not applicable; density (at 20 °C): 1 g/cm^3^; solubility in water: soluble; pH: 6.05
Tap water	Ammonium (NH^4+^): <0.05 mg/L; total hardness (°dH): 8.18; calcium (Ca^2+^): 58.44 mg/L; magnesium (Mg^2+^): 35.44 mg/L; nitrites (NO^2−^): <0.033 mg/L; nitrates (NO^3−^): 5.02 mg/L; free residual chlorine: 0.33 mg/L; oxidability (permanganate index method): 1.29 mg/L; pH = 7.66
Nutrient solution A (sol. A) (Liquid fertilizer for balcony flowers, produced by AGRO CS, Lucenec, Slovakia [69])	Synthetic product that was used according to the manufacturer’s instructions: 7.5 mL diluted in 1000 mL water; total nitrogen: 3.6%; nitric nitrogen (NO_3_^−^): 1.8%; ammoniacal nitrogen (NH_4_^+^): 1.8%; phosphorus (P_2_O_5_): 2.3%; potassium (K_2_O): 2.7%; pH = 5.40 [69]
Nutrient solution B (sol. B) (Biopon, natural biohumus for vegetables and greens flowers, produced by Bros Sp. z o.o. sp. k., Poznan, Poland [69])	Organic product, 100% natural (made from vermicompost) that was used according to the manufacturer’s instructions: 60 mL diluted in 1000 mL water; total nitrogen: 0.02%; phosphorus (P_2_O_5_): 0.02%; potassium (K_2_O): 0.05%; micronutrients: copper (0.25 mg/L), zinc (1.20 mg/L), iron (40 mg/L), manganese (2.1 mg/L); organic matter: 40.0%; pH = 7.45 [69]

## Data Availability

The original contributions presented in the study are included in the article, further inquiries can be directed to the corresponding author.

## References

[1] Azeem M.K., Islam A., Khan R.U., Rasool A., Qureshi M.A.R., Rizwan M., Sher F., Rasheed T. (2023). Eco-friendly three-dimensional hydrogels for sustainable agricultural applications: Current and future scenarios. Polym. Adv. Technol..

[2] Tariq Z., Iqbal D.N., Rizwan M., Ahmad M., Faheem M., Ahmed M. (2023). Significance of biopolymer-based hydrogels and their applications in agriculture: A review in perspective of synthesis and their degree of swelling for water holding. RSC Adv..

[3] Jacob P., Ruiz Cantu L., Pearce A., He Y., Lentz J., Moore J., Machado F., Rivers G., Apebende E., Romero Fernandez M. (2021). Poly (glycerol adipate) (PGA) backbone modifications with a library of functional diols: Chemical and physical effects. Polymer.

[4] Chronopoulou L., Cacciotti I., Amalfitano A., Di Nitto A., D’Arienzo V., Nocca G., Palocci C. (2020). Biosynthesis of innovative calcium phosphate/hydrogel composites: Physicochemical and biological characterization. Nanotechnology.

[5] Santhamoorthy M., Kim S.-C. (2023). Dual pH- and Thermo-Sensitive Poly(N-isopropylacrylamide-co-allylamine) Nanogels for Curcumin Delivery: Swelling–Deswelling Behavior and Phase Transition Mechanism. Gels.

[6] Mostafavi A., Quint J., Russell C., Tamayol A., Vrana N.E., Knopf-Marques H., Barthes J. (2020). Nanocomposite hydrogels for tissue engineering applications. Biomaterials for Organ and Tissue Regeneration. New Technologies and Future Prospects.

[7] Zhao Y., Song S., Ren X., Zhang J., Lin Q., Zhao Y. (2022). Supramolecular Adhesive Hydrogels for Tissue Engineering Applications. Chem. Rev..

[8] Guilherme M., Aouada F., Fajardo A., Martins A., Paulino A., Davi M., Rubira A., Muniz E. (2015). Superabsorbent hydrogels based on polysaccharides for application in agriculture as soil conditioner and nutrient carrier: A review. Eur. Polym. J..

[9] Guo Y., Bae J., Fang Z., Li P., Zhao F., Yu G. (2020). Hydrogels and Hydrogel-Derived Materials for Energy and Water Sustainability. Chem. Rev..

[10] Guo Y., Bae J., Zhao F., Yu G. (2019). Functional Hydrogels for Next-Generation Batteries and Supercapacitors. Trends Chem..

[11] Hennink W.E., van Nostrum C.F. (2002). Novel crosslinking methods to design hydrogels. Adv. Drug Deliv. Rev..

[12] Peppas N.A., Hilt J.Z., Khademhosseini A., Langer R. (2006). Hydrogels in biology and medicine: From molecular principles to bionanotechnology. Adv. Mater..

[13] El-Naggar A.A. (2016). Radiation synthesis of superabsorbent hydrogels based on carboxymethyl cellulose/sodium alginate for absorbent of heavy metal ions from waste water. J. Thermoplast. Compos..

[14] Pourjavadi A., Harzandi A., Hosseinzadeh H. (2004). Synthesis of a novel polysaccharide-based superabsorbent hydrogel via graft copolymerization of acrylic acid onto kappa-carrageenan in air. Eur. Polym. J..

[15] Pourjavadi A., Ayyari M., Amini-Fazl A. (2008). Taguchi optimized synthesis of collagen-g-poly (acrylic acid)/kaolin composite superabsorbent hydrogel. Eur. Polym. J..

[16] Bashir S., Hina M., Iqbal J., Rajpar A.H., Mujtaba M.A., Alghamdi N.A., Wageh S., Ramesh K., Ramesh S. (2020). Fundamental Concepts of Hydrogels: Synthesis, Properties, and Their Applications. Polymers.

[17] Palanivelu S.D., Armir N.A.Z., Zulkifli A., Hair A.H.A., Salleh K.M., Lindsey K., Che-Othman M.H., Zakaria S. (2022). Hydrogel Application in Urban Farming: Potentials and Limitations—A Review. Polymers.

[18] Qureshi M.A., Nishat N., Jadoun S., Ansari M.Z. (2020). Polysaccharide based superabsorbent hydrogels and their methods of synthesis: A review. Carbohydr. Polym. Technol. Appl..

[19] Sikdar P., Uddin M.M., Dip T.M., Islam S., Hoque M.S., Dhar A.K., Wu S. (2021). Recent Advances in the Synthesis of Smart Hydrogels. Mater. Adv..

[20] Yang J., Rao L., Wang Y., Zhao Y., Liu D., Wang Z., Fu L., Wang Y., Yang X., Li Y. (2022). Recent Advances in Smart Hydrogel Prepared by Ionizing Radiation Technology for Biomedical Applications. Polymers.

[21] Trivedi J., Chourasia A. (2023). Sodium Salt of Partially Carboxymethylated Sodium Alginate-Graft-Poly(Acrylonitrile): II Superabsorbency, Salt Sensitivity and Swelling Kinetics of Hydrogel, H-Na-PCMSA-g-PAN. Gels.

[22] Yang Z., Zhai X., Li M., Li Z., Shi J., Huang X., Zou X., Yan M., Qian W., Gong Y. (2022). Saccharomyces cerevisiaeincorporated and sucrose-rich sodium alginate film: An effective antioxidant packaging film for longan preservation. Int. J. Biol. Macromol..

[23] Sramkova P., Kucka J., Kronekova Z., Lobaz V., Slouf M., Micusík M., Josef Sepitka J., Kleinova A., Chorvat D., Mateasik A. (2023). Electron beam irradiation as a straightforward way to produce tailorable non-biofouling poly(2-methyl-2-oxazoline) hydrogel layers on different substrates. Appl. Surf. Sci..

[24] Stachowiak N., Kowalonek J., Kozlowska J., Burkowska-But A. (2023). Stability Studies, Biodegradation Tests, and Mechanical Properties of Sodium Alginate and Gellan Gum Beads Containing Surfactant. Polymers.

[25] Zhao L., Zhou Y., Zhang J., Liang H., Chen X., Tan H. (2023). Natural Polymer-Based Hydrogels: From Polymer to Biomedical Applications. Pharmaceutics.

[26] Kowalski G., Witczak M., Kuterasinski L. (2024). Structure Effects on Swelling Properties of Hydrogels Based on Sodium Alginate and Acrylic Polymers. Molecules.

[27] Tang S., Zhao Y., Wang H., Wang Y., Zhu H., Chen Y., Chen S., Jin S., Yang Z., Li P. (2018). Preparation of the Sodium Alginate-g-(Polyacrylic Acid-co-Allyltrimethylammonium Chloride) Polyampholytic Superabsorbent Polymer and Its Dye Adsorption Property. Mar. Drugs.

[28] Ismaeilimoghadam S., Jonoobi M., Hamzeh Y., Danti S. (2022). Effect of Nanocellulose Types on Microporous Acrylic Acid/Sodium Alginate Super Absorbent Polymers. J. Funct. Biomater..

[29] Abouzeid R.E., Salama A., El-Fakharany E.M., Guarino V. (2022). Mineralized Polyvinyl Alcohol/Sodium Alginate Hydrogels Incorporating Cellulose Nanofibrils for Bone and Wound Healing. Molecules.

[30] Elyashevich G., Rosova E., Zoolshoev Z., Saprykina N., Kuryndin I. (2023). Reversibility of Swelling, pH Sensitivity, Electroconductivity, and Mechanical Properties of Composites Based on Polyacrylic Acid Hydrogels and Conducting Polymers. J. Compos. Sci..

[31] Bai M., Wilske B., Buegger F., Esperschutz J., Bach M., Frede H.G., Breuer L. (2015). Relevance of nonfunctional linear polyacrylic acid for the biodegradation of superabsorbent polymer in soils. Environ. Sci. Pollut. Res..

[32] Caykara T., Demirci S., Eroglu M.S., Guven O. (2005). Poly(ethylene oxide) and its blends with sodium alginate. Polymer.

[33] Kondo T., Sawatari C. (1994). Intermolecular hydrogen bonding in cellulose/poly(ethylene oxide) blends: Thermodynamic examination using 2,3-di-O- and 6-O-methylcelluloses as cellulose model compounds. Polymer.

[34] Erizal E., Wikanta T. (2011). Synthesis of polyethylene oxide (peo)–chitosan hydrogel prepared by gamma radiation technique. Indones. J. Chem..

[35] Craciun G., Calina I.C., Demeter M., Scarisoreanu A., Dumitru M., Manaila E. (2023). Poly(Acrylic Acid)-Sodium Alginate Superabsorbent Hydrogels Synthesized by Electron Beam Irradiation Part I: Impact of Initiator Concentration and Irradiation Dose on Structure, Network Parameters and Swelling Properties. Materials.

[36] Manaila E., Demeter M., Calina I.C., Craciun G. (2023). NaAlg-g-AA Hydrogels: Candidates in Sustainable Agriculture Applications. Gels.

[37] ALSamman M.T., Sánchez J. (2022). Chitosan- and Alginate-Based Hydrogels for the Adsorption of Anionic and Cationic Dyes from Water. Polymers.

[38] Salvbaroy Polar Iceberg Water https://svalbardi.com/blogs/water/distilled.

[39] Lin H., Zhou J., Yingde C., Gunasekaran S. (2010). Synthesis and Characterization of pH- and Salt-Responsive Hydrogels Based on Etherificated Sodium Alginate. J. Appl. Polym. Sci..

[40] Hajime M., Masato M., Mitsuru S. (2001). Ion-specific swelling of hydrophilic polymer gels. Polymer.

[41] da Silva Fernandes R., Tanaka F.N., de Moura M.R., Aouada F.A. (2019). Development of alginate/starch-based hydrogels crosslinked with different ions: Hydrophilic, kinetic and spectroscopic properties. Mater. Today Commun..

[42] Gierszewska M., Ostrowska-Czubenko J., Chrzanowska E. (2018). pH-responsive chitosan/alginate polyelectrolyte complex membranes reinforced by tripolyphosphate. Eur. Polym. J..

[43] Chen X.X., Shan G.G., Huang J., Huang Z.Z., Weng Z.Z. (2004). Synthesis and properties of acrylic-based superabsorbent. J. Appl. Polym. Sci..

[44] Flory P.J., Rehner J. (1943). Statistical mechanics of cross-linked polymer networks II. Swelling. J. Chem. Phys..

[45] Wu X., Huang X., Zhu Y., Li J., Hoffmann M.R. (2020). Synthesis and application of superabsorbent polymer microspheres for rapid concentration and quantification of microbial pathogens in ambient water. Sep. Purif. Technol..

[46] Ciftbudak S., Orakdogen N. (2023). Correlation between effective charge density and crosslinking efficiency of dicarboxylic acid containing highly anionic networks. Polymer.

[47] Böni L.J., Zurflüh R., Baumgartner M.E., Windhab E.J., Fischer P., Kuster S., Rühs P.A. (2018). Effect of ionic strength and seawater cations on hagfish slime formation. Sci. Rep..

[48] Kalantari S., Hatami S., Ardalan M.M., Alikhani H.A., Shorafa M. (2010). The effect of compost and vermicompost of yard leaf manure on growth of corn. Afr. J. Agric. Res..

[49] Lv Q., Wu M., Shen Y. (2019). Enhanced swelling ratio and water retention capacity for novel superabsorbent hydrogel. Colloid. Surface A.

[50] Yavari N., Saeid Azizian S. (2022). Mixed diffusion and relaxation kinetics model for hydrogels swelling. J. Mol. Liq..

[51] Madduma-Bandarage U.S.K., Madihally S.V. (2021). Synthetic hydrogels: Synthesis, novel trends, and applications. J. Appl. Polym. Sci..

[52] Mondal S., Das S., Nandi A.K. (2020). A review on recent advances in polymer and peptide hydrogels. Soft Matter.

[53] Samateh M., Pottackal N., Manafirasi S., Vidyasagar A., Maldarelli C., John G. (2018). Unravelling the secret of seed-based gels in water: The nanoscale 3D network formation. Sci. Rep..

[54] Kankala R.K., Wang S.-B., Chen A.-Z., Zhang Y.S., Conde J. (2018). Self-Assembled Nanogels: From Particles to Scaffolds and Membranes. Handbook of Nanomaterials for Cancer Theranostics.

[55] Gao S., Jiang G., Li B., Han P. (2018). Effects of high-concentration salt solutions and pH on swelling behavior of physically and chemically cross-linked hybrid hydrophobic association hydrogels with good mechanical strength. Soft Mater..

[56] Flory P.J. (1953). Principles of Polymer Chemistry.

[57] Pourjavadi A., Kurdtabar M., Ghasemzadeh H. (2008). Salt-and pH-resisting collagen based highly porous hydrogel. Polym. J..

[58] Baker J.P., Hong L.H., Blanch H.W., Prausnitz J.M. (1994). Effect of initial total monomer concentration on the swelling behavior of cationic acrylamide based hydrogels. Macromolecules.

[59] Li X., Wang Y., Li D., Shu M., Shang L., Xia M., Huang Y. (2021). High-strength, thermosensitive double network hydrogels with antibacterial functionality. Soft Matter.

[60] Jovanovic J., Adnadjevic B., Kostic A. (2010). The effects of the pH value of the swelling medium on the kinetics of the swelling of a poly (acrylic acid) hydrogel. J. Appl. Polym. Sci..

[61] Ostrowska-Czubenko J., Gierszewska M., Pieróg M. (2015). pH-responsive hydrogel membranes based on modified chitosan: Water transport and kinetics of swelling. J. Polym. Res..

[62] Alizadeh M., Abbasi F., Farahi M., Jalili K. (2012). Silicone-based hydrogels prepared by interpenetrating polymer network synthesis: Swelling properties and confinements effects on the formation kinetics. J. Appl. Polym. Sci..

[63] Tanaka T. (1981). Gels. Sci. Am..

[64] Peters A., Candau S. (1988). Kinetics of swelling of spherical and cylindrical gels. Macromolecules.

[65] Singh T., Singhal R. (2012). Poly (acrylic acid/acrylamide/sodium humate) superabsorbent hydrogels for metal ion/dye adsorption: Effect of sodium humate concentration. J. Appl. Polym. Sci..

[66] Kim S.J., Lee K.J., Kim I.Y., Kim S.I. (2003). Swelling kinetics of interpenetrating polymer hydrogels composed of poly (vinyl alcohol)/chitosan. J. Macromol. Sci. A.

[67] Schott H. (1992). Swelling kinetics of polymers. J. Macromol. Sci. B.

[68] Schott H. (1992). Kinetics of swelling of polymers and their gels. J. Pharm. Sci..

[69] Kostic A., Adnadjevic B., Popovic A., Jovanovic J. (2007). Comparison of the swelling kinetics of a partially neutralized poly (acrylic acid) hydrogel in distilled water and physiological solution. J. Serb. Chem. Soc..

[70] Skrzypczaka D., Mikulaa K., Kossińskab N., Widerab B., Warchoła J., Moustakasc K., Chojnackaa K., Witek-Krowiakb A. (2020). Biodegradable hydrogel materials for water storage in agriculture—Review of recent research. Desalination Water Treat..

[71] Costa P., Sousa Lobo J.M. (2001). Modeling and comparison of dissolution profiles. Eur. J. Pharm. Sci..

[72] Peppas N.A., Bures P., Leobandung W., Ichikawa H. (2000). Hydrogels in pharmaceutical formulations. Eur. J. Pharm. Biopharm..

[73] Brazel C.S., Peppas N.A. (1999). Mechanisms of solute and drug transport in relaxing, swellable, hydrophilic glassy polymers. Polymer.

[74] Erizal E., Sudirman S., Budianto E., Mahendra A., Yudianti R. (2013). Radiation Synthesis of Superabsorbent Poly(acrylamide-co-acrylic acid)-Sodium Alginate Hydrogels. Adv. Mater. Res..

[75] Manaila E., Craciun G., Ighigeanu D., Calina I.C. (2023). Sodium Alginate-g-acrylamide/acrylic Acid Hydrogels Obtained by Electron Beam Irradiation for Soil Conditioning. Int. J. Mol. Sci..

[76] Karadag E., Saraydin D. (2002). Swelling studies of super water retainer acrylamide/crotonic acid hydrogels crosslinked by trimethylolpropane triacrylate and 1,4-butanediol dimethacrylate. Polym. Bull..

[77] Karadag E., Saraydin D., Sahiner N., Güven O. (2001). Radiation induced acrylamide/citric acid hydrogelas and their swelling behaviors. J. Macromol. Sci. A.

[78] Niu C., Li X., Wang Y., Liu X., Shi J., Wang X. (2019). Design and performance of a poly(vinyl alcohol)/silk fibroin enzymatically crosslinked semiinterpenetrating hydrogel for a potential hydrophobic drug delivery. RSC Adv..

[79] James J.D., Ludwick J.M., Wheeler M.L., Oyen M.L. (2020). Compressive failure of hydrogel spheres. J. Mater. Res..

[80] Karadag E., Uzum O.B., Saraydin D. (2002). Swelling equilibria and dye adsorption studies of chemically crosslinked superabsorbent acrylamide/maleic acid hydrogels. Eur. Polym. J..

[81] Jabbari E., Nozari S. (2000). Swelling behaviour of acrylic acid hydrogels prepared by c-radiation crosslinking of polyacrylic acid in aqueous solution. Eur. Polym. J..

[82] Espert A., Vilaplana F., Karlsson S. (2004). Comparison of water absorption in natural cellulosic fibres from wood and one-year crops in polypropylene composites and its influence on their mechanical properties. Compos. Part A Appl. Sci. Manuf..

[83] Kumar K.A.A., Sreekala M.S., Arun S. (2012). Studies on Properties of Bio-Composites from Ecoflex/Ramie Fabric-Mechanical and Barrier Properties. J. Biomater. Nanobiotechnol..

[84] Ehi I.P., Bidemi J.K., Yahaya L.E. (2016). Kinetic Studies on Water Absorption properties of Cocoa-pod Epoxy Composites. Iran. (Iran.) J. Energy Environ..

